# *Emoji* as promising tools for emotional evaluation in orthodontics

**DOI:** 10.1186/s40510-022-00418-3

**Published:** 2022-07-18

**Authors:** Guido Artemio Marañón-Vásquez, Lucianne Cople Maia, Luísa Schubach da Costa Barreto, Mariana Farias da Cruz, Lucas Alves Jural, Mônica Tirre de Souza Araújo, Matheus Melo Pithon

**Affiliations:** 1 Department of Pediatric Dentistry and Orthodontics, School of Dentistry, Federal University of Rio de Janeiro, Cidade Universitária da Universidade Federal Do Rio de Janeiro, Rua. Prof. Rodolpho Paulo Rocco, 325, Rio de Janeiro, RJ 21941-617 Brazil; 2 Department of Health I, School of Dentistry, Southwest Bahia State University, Av. José Moreira Sobrinho, s/n, Jequiezinho, Jequié, BA 45206190 Brazil

**Keywords:** Emotions, Expressed emotion, Malocclusion, Surveys and questionnaires, Nonverbal communication

## Abstract

**Background:**

*Emoji* are pictograms frequently used in social networks capable of expressing emotions. These tools can provide insights into people's behavior that could not be obtained with the use of textual communication. Recently, *emoji* have been introduced to various research fields as successful alternatives to word-based questionnaires for measure emotional responses. The objective of this study was to preliminarily evaluate the discriminating ability and relationship of these tools with different occlusal conditions/malocclusions.

**Methods:**

Online surveys were applied to adult individuals (*n* = 201; mean age = 27.4 ± 5.7; 37.3% males, 62.7% females). Subjects issued acceptance scores (10-point scale) and expressed their emotional status using a 30-*emoji* list in relation to nine occlusal conditions: C1–crowding, C2–anterior open bite, C3–interincisal diastema, C4–increased overjet + deep bite (Class II div. 1), C5–anterior crossbite (Class III), C6–ideal occlusion, C7–unilateral posterior crossbite, C8–anterior open bite plus bilateral posterior crossbite plus crowding, and C9–deep bite (Class II div. 2). Cochran's Q and McNemar tests were used to compare the frequencies of choice of *emoji* between conditions. Correspondence analyses were applied to assess the association between occlusal conditions and *emoji*. Kendall's correlation coefficient was calculated to evaluate the relationship between mean acceptance scores and frequency counts of each *emoji*.

**Results:**

The frequency of choice between conditions showed a significant difference for 25 of the 30 *emoji* (*P* < 0.05), indicating an adequate discriminating ability of these tools. *Emoji* were grouped predominantly based on their emotional valence (positive/negative) and arousal/activation (high/low). Positive *emoji* were associated with the most accepted conditions (*i.e.*, C6, C3), while negative *emoji* with the most rejected ones (*i.e.*, C8, C1, C2). Although only weak, positive correlations between acceptance and positively valenced *emoji*, and negative correlations between acceptance and negatively valenced *emoji* were observed (*P* < 0.05).

**Conclusions:**

*Emoji* have an adequate discriminatory ability and would allow determining emotional profiles in the face of different occlusal conditions. Further research is necessary to consolidate the use of these tools in an instrument that allows measuring emotional responses.

**Supplementary Information:**

The online version contains supplementary material available at 10.1186/s40510-022-00418-3.

## Background

Emotions can be measured in different ways [1]. Self-report is perhaps the most widely used procedure for evaluating currently experienced emotions. Word-based questionnaires have been commonly used in several research fields for this purpose. However, their questionable ecological validity [2, 3], the ambiguity between selected emotion words and the actual emotion experienced [4], poor understanding of terms listed in questionnaires [2], and the inability to capture intuitive and automatic emotional evoked associations [3], have prompted the need to develop non-verbal methods.

*Emoji* are pictograms frequently used in social networks capable of expressing emotions [5], in the way they would be presented in a face-to-face interaction [6]. These tools have the ability to provide insights into people's behavior that could not be obtained with the use of textual communication [7]. Different areas of knowledge have ventured into *emoji* research, such as computer science, communication, marketing, behavioral science, linguistics, psychology, education, and even medicine [8]. Interestingly, previous research on consumer food preferences introduced the use of *emoji* as a successful alternative to word-based surveys to measure emotional responses [9–11].

In dentistry, as far as we know, *emoji* have only been used in the development of scales to assess, for example, anxiety or pain in pediatric patients [12, 13]. There are no investigations using *emoji* as tools for assessing emotional profiles in oral research. Since it has been shown that malocclusions can affect different holistic aspects of health such as the emotional dimension [14–16], we consider these conditions suitable to test the use of *emoji.* The main objective of the present study was to preliminarily evaluate the discriminating ability and relationship of these tools with different occlusal conditions/malocclusions. We consider this research as the first necessary step towards the subsequent development of an *emoji*-based instrument that allows measuring the emotional response related to these conditions.

## Materials and methods

The protocol of the present study was approved by the research ethics committee of the Clementino Fraga Filho University Hospital ((# 17,557,319.4.0000.5257). Digital informed consent was obtained from all participants before the start of the survey.

### Participants

Adult individuals (≥ 18 years) were recruited through calls via researchers' social networks, for a one-week period. Dentists, dental assistants, and dental students were not included. Of 303 subjects who showed interest in participating, 292 were eligible. Only those participants who fully completed the questionnaires sent were included in the study (*n* = 201; mean age = 27.4 ± 5.7; 37.3% males, 62.7% females).

### Images of occlusal conditions/malocclusions

Frontal intraoral pretreatment photographs of orthodontic patients were scrutinized from records of the private clinic of one of the researchers (M.M.P.), in order to identify specific malocclusions. Informed consent was obtained from patients whose photographs were chosen.

The selected photographs had been acquired using cheek and lip separators for a complete exposure of the dental arches. In order to show the occlusal conditions in a more familiar context to the participants, these images were embedded into a smile frame using Adobe Photoshop CS6 (Adobe Systems Inc., San José, CA, USA). Initially, in a photograph of a symmetrical smile, the area of the teeth and gums was selected and removed, keeping only the lips and extraoral regions. This image (top layer) was then superimposed on the photographs of the occlusal conditions (background layers). Using the brush tool, subtle gradients were created in the joining regions between both images; and, subsequently, adjustments were made in contrast, temperature and saturation in order to have a more realistic appearance. Finally, the new images were cut in a standardized way in a 3:4 ratio. No editing was performed to modify the characteristics of the occlusal conditions.

**Fig. 1:**
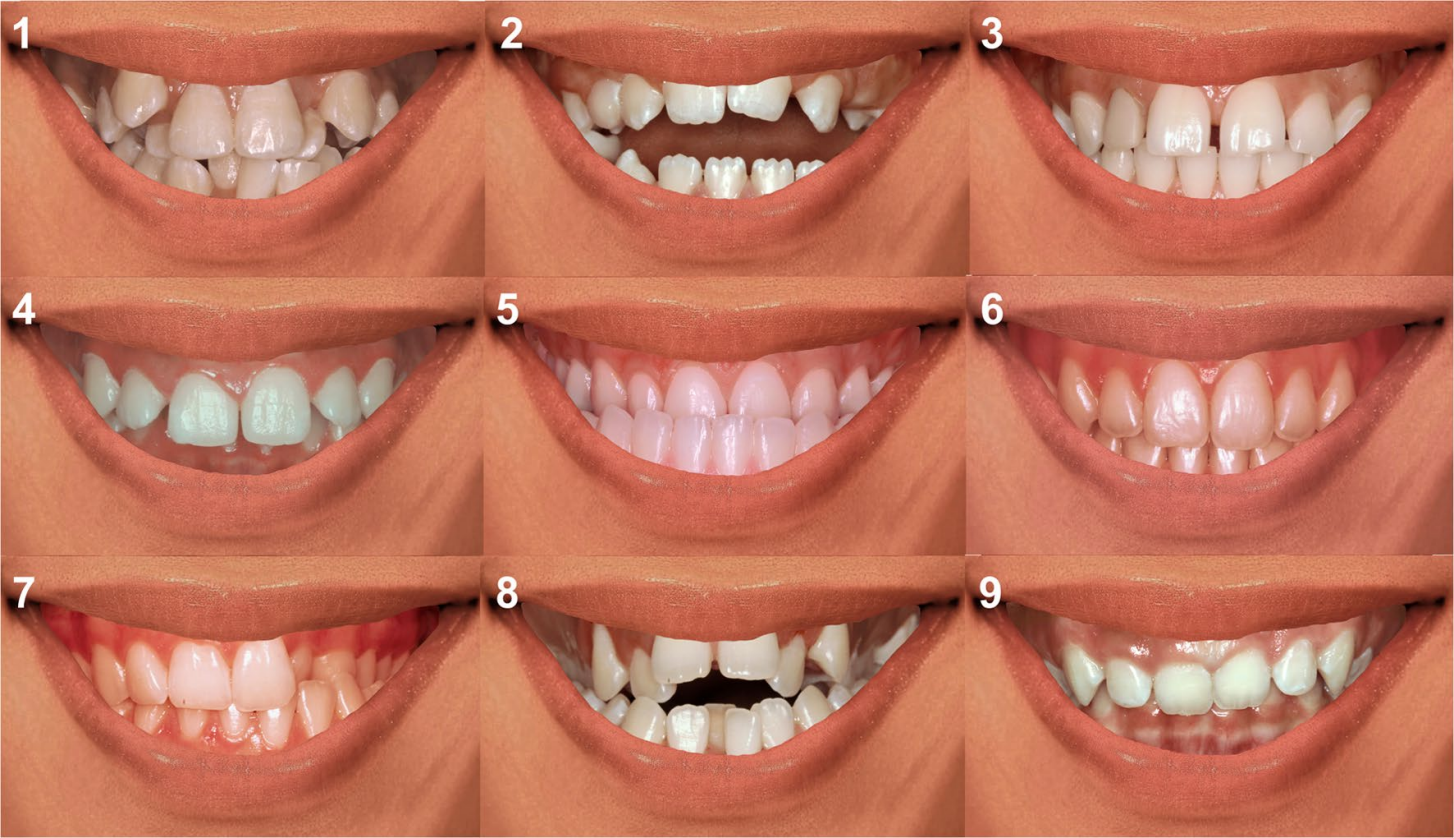
Oral conditions presented in questionnaires. C1—crowding, C2—anterior open bite, C3—interincisal diastema, C4—increased overjet and deep bite (Class II division 1), C5—anterior crossbite (Class III), C6—ideal occlusion, C7—unilateral posterior crossbite, C8—anterior open bite plus bilateral posterior crossbite plus crowding, and C9—deep bite (Class II division 2)

The selected conditions were randomly ordered for presentation in questionnaires (Fig. 1), as follows: C1–crowding, C2–anterior open bite, C3–interincisal diastema, C4–increased overjet and deep bite (Class II division 1), C5–anterior crossbite (Class III), C6–ideal occlusion, C7–unilateral posterior crossbite, C8–anterior open bite plus bilateral posterior crossbite plus crowding, and C9–deep bite (Class II division 2).

### Emoji selection

**Fig. 2.**
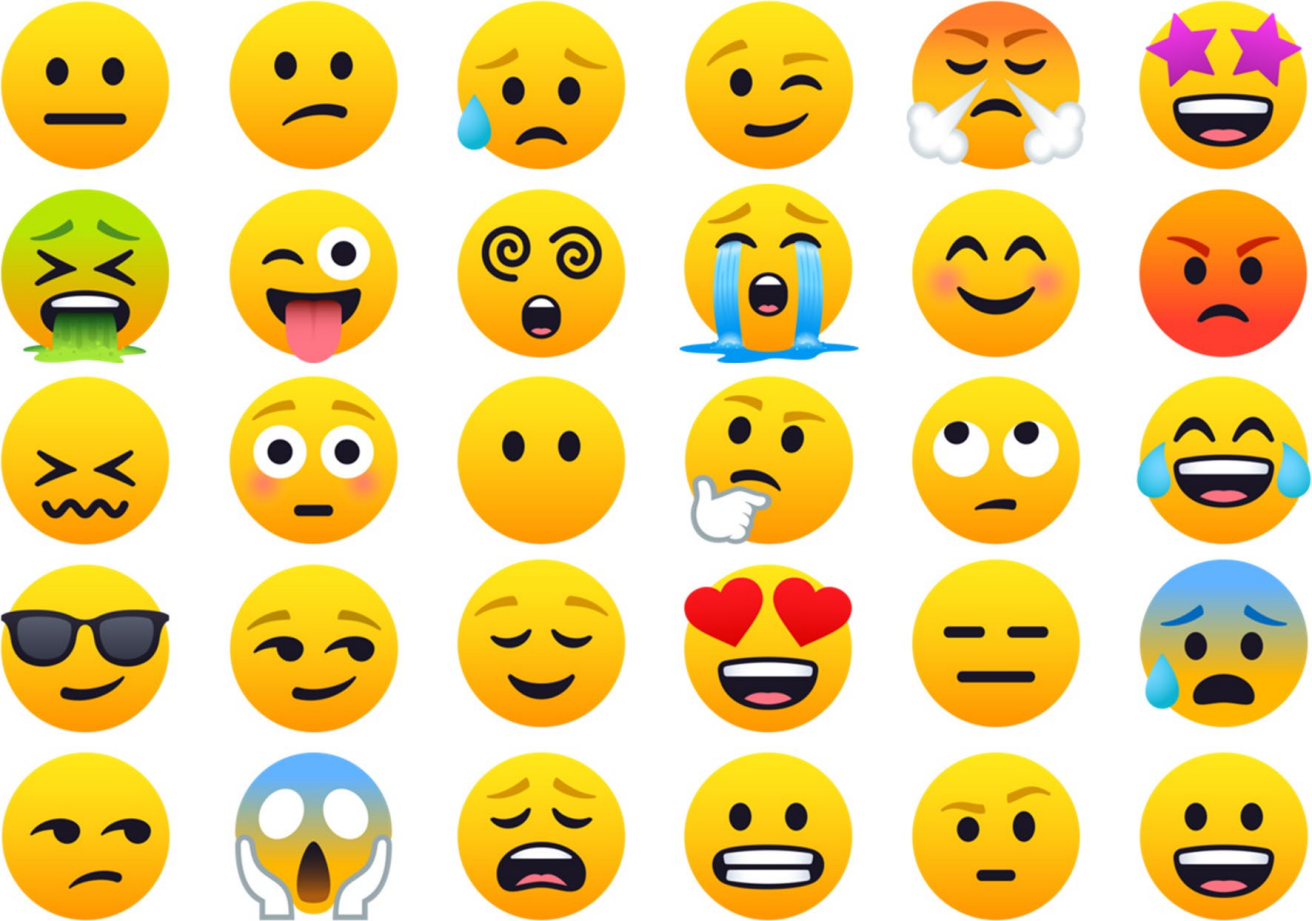
30-*emoji* list presented in questionnaires

Most widely used *emoji* (Smileys & People category from https://emojipedia.org/) were pre-selected based on the site https://emojitracker.com/ (real-time *emoji* use on Twitter). Irrelevant *emoji* were excluded (*e.g.*,  ). In the case of *emoji* with similar meanings (*e.g.*,  ), the one with the highest frequency of use was maintained. Thirty *emoji* from JoyPixels version 4.5 (https://www.joypixels.com/; Free License Agreement) were finally selected and randomly ordered for presentation in the questionnaires (Fig. 2). Based on the meanings provided on http://emojipedia.org/ (Additional file 3: Table S1) and information from previous research [11, 17–20], *emoji* were pre-classified according to their emotional valence in positive, negative, and neutral.

### Questionnaire

Online questionnaires were developed on SurveyMonkey ® platform (https://it.surveymonkey.com/) and were sent through the social network by which participants were contacted. Structure of the forms was divided into four sections. The first one included questions on socio-demographic data (sex and age). In the second section, images of occlusal conditions were presented together (Fig. 1) and individuals were consulted about which condition was the most accepted and which was the most rejected by them. Subsequently, participants issued scores on their overall acceptance (liking) independently for each condition using a 10-point scale (1 = they disliked the condition very much, 10 = they liked it very much). The third section comprised evaluations using the 30-*emoji* list (Fig. 2). Participants were asked to select all *emoji* that they considered represented their emotions after observing each condition separately (*i.e.*, as many *emoji* as they wanted). The final section of the form included questions about smile esthetics and bite self-perception (10-point scale; 1 = extremely bad, 10 = excellent), orthodontic treatment need (esthetic component [EC] of the IOTN index [21, 22]: “none/little need” [EC = 1–4], “moderate need” [EC = 5–7], “great/extreme need” [EC = 8–10]), previous experience of orthodontic treatment (“in treatment”, “received treatment”, “did not receive treatment”) and, daily frequency of use of *emoji* (“never”, “rarely”, “sometimes”, “often”, “always”).

### Data analysis

A generalized mixed model was implemented to assess overall acceptance scores issued by participants for the nine malocclusions. The occlusal conditions were considered as fixed source of variation and the examiners as random effect. Bonferroni post hoc test was used for subsequent pairwise comparisons.

To assess the discriminating ability of *emoji*, frequencies of choice of these tools for each of the oral conditions were compared using the Cochran's Q test. In the presence of significant differences, post hoc comparisons were performed using the McNemar test. When appropriate, binomial logistic regression models were used to examine whether *emoji* choice was influenced by the variables age, sex, smile esthetics and bite self-perception, previous experience of orthodontic treatment, and daily frequency of use of *emoji*. The variable orthodontic treatment need was not included in the regression model since 99.5% of participants indicated having small need for treatment. Regression analyses were only applied when the frequency of choosing an *emoji* was ≥ 10% for a certain condition.

Correspondence analysis was conducted (1) to summarize and visualize the large data set of the variables ‘occlusal condition’ and ‘*emoji*’ in simplified two-dimension plots, and (2) to evaluate the relationship between categories of both evaluated variables. Analysis was based on a frequency table that had malocclusions in the rows and total frequencies for *emoji* in the columns. The mean overall acceptance for each condition was considered as a supplementary variable. Multiple correspondence analysis was also performed taking into account the individual responses of participants. Independent evaluations for each occlusal condition were arranged in the rows, and responses on the choice of each *emoji* in the columns. Oral conditions and overall acceptance were considered as supplementary categories.

Kendall’s Tau-b correlation coefficients were calculated to evaluate the relationship between the mean overall acceptance and frequency counts for each *emoji*. Values recommended by Cohen were used to determine strength of correlations (weak, *r* < 0.3; moderate, 0.3 ≤ *r* ≤ 0.5; strong, r > 0.5) [23]. Having the assumption that positive emotional responses correspond to a greater liking of a certain stimulus, this analysis was carried out to evaluate/confirm the emotional valence of *emoji*, and to interpret their arrangement in the factorial maps generated by the correspondence analyses.

All the above-mentioned analyses were performed using free access software BioEstat 5.0 (Belém, PA, Brazil) and Jamovi (version 1.2). The significance level adopted was 5%. Simple and multiple correspondence analyses were performed in R 4.0.3 using the R-packages FactoMineR and factoextra.

## Results

There was a significant difference in the overall acceptance of the oral conditions (*P* < 0.001). The most accepted ones were C6 and C3 (Additional file 1: Fig. S1), which presented mean overall acceptances of 7.15 (95% CI: 6.71, 7.62) and 4.85 (95% CI: 4.51, 5.22), respectively. On the other hand, the most rejected conditions were C8, C1, and C2 (Additional file 1: Fig. S1), with mean overall acceptances of 1.24 (95% CI: 1.09, 1.40), 1.39 (95% CI: 1.25, 1.55) and 1.58 (95% CI: 1.41, 1.77), respectively. Mean counts of overall acceptance for all conditions are shown in Table 1.

**Table 1:** Mean counts of overall acceptance for oral conditions

	Mean count (SE)	95% CI
C1	1.39 (0.08)	(1.25, 1.55)^a^
C2	1.58 (0.09)	(1.41, 1.77)^a^
C3	4.85 (0.18)	(4.51, 5.22)^b^
C4	3.76 (0.15)	(3.47, 4.07)^c^
C5	2.93 (0.13)	(2.68, 3.19)^df^
C6	7.15 (0.23)	(6.71, 7.62)^e^
C7	3.42 (0.14)	(3.15, 3.72)^cd^
C8	1.24 (0.08)	(1.09, 1.40)^a^
C9	2.60 (0.12)	(2.38, 2.85)^f^
*P*-value	< 0.001	

SE—standard error, CI—confidence interval, C1—crowding, C2—anterior open bite, C3—interincisal diastema, C4—increased overjet and deep bite (Class II division 1), C5—anterior crossbite (Class III), C6—ideal occlusion, C7—unilateral posterior crossbite, C8—anterior open bite plus bilateral posterior crossbite plus crowding, C9—deep bite (Class II division 2)

^a,^^b,c,d,e,f^ Different superscript letters indicate difference between conditions

On average, participants used 2.06 *emoji* out of 30 available (between 1 to 12 *emoji* per response were indicated), ranging from 1.6 (for C3) to 2.8 (for C8). The frequency of choice of each *emoji* ranged from 0.72% (  ) to 23.9% (  ). The most used *emoji* (frequency of use > 10%) were  ; while the least used (≤ 1%) were  . Significant differences in the frequency of use were evidenced for 25 of the 30 *emoji* (*P* < 0.05), indicating their ability to discriminate between the conditions presented (Table 2). A consistent pattern of contribution of any of the explanatory variables tested on the frequency of emoji choice was not evidenced (only isolated associations were observed, Additional file 4: Table S2).

**Table 2:** Frequency (%) of choice of each *emoji* among the oral conditions

*Emoji*	Oral condition									*P*-value
	C1	C2	C3	C4	C5	C6	C7	C8	C9	
*Positive valence*										
	0.0	1.5^a^	13.4^b^	7.5^c^	2.0^a^	25.4^d^	1.5^a^	0.0	4.5^ac^	< 0.001
	0.0	0.0	2.5^a^	0.0	0.5^a^	12.9^b^	0.0	0.0	0.0	< 0.001
	0.0	1.0^a^	6.5^b^	3.0^ab^	2.0^a^	6.5^b^	1.0^a^	1.0^a^	2.0^a^	< 0.001
	0.0	0.5^a^	11.9^b^	7.0^b^	2.5^a^	29.4^c^	2.0^a^	0.0	2.5^a^	< 0.001
	2.0	1.5	1.0	1.5	3.5	0.5	0.5	1.5	1.0	0.230
	0.0	0.0	1.0^a^	0.5^a†^	0.5^a†^	6.5^b^	0.0	0.0	0.5^a^	< 0.001
	0.5^a^	2.0^ab^	2.0^ab^	2.5^ab^	1.0^a^	5.0^b^	2.0^ab^	0.0	2.5^ab^	0.019
	0.5^a^	0.0	13.9^b^	8.5^c^	1.0^a^	17.9^b^	1.0^a^	0.0	2.0^a^	< 0.001
	0.0	0.0	2.0^a^	0.5^a^	0.0	10.9^b^	0.0	0.0	0.0	< 0.001
	0.5^a^	1.0^ac^	10.0^b^	3.0^c^	2.0^ac^	23.4^d^	1.0^ac^	1.0^ac^	2.0^ac^	< 0.001
Negative valence										
	29.9^ad^	28.9^ad^	19.4^b^	19.9^b^	24.9^ab^	2.5^c^	30.8^a^	22.9^bd^	29.9^a^	< 0.001
	33.8^ae^	36.3^a^	7.5^b^	11.4^b^	19.9^c^	2.5^d^	23.4^cf^	38.3^a^	26.9^ef^	< 0.001
	1.5^ab^	2.0^ab^	0.0	1.5^ab^	0.5^a^	0.5^a^	0.5^a^	3.5^b^	1.5^ab^	0.024
	17.9^a^	7.0^b^	0.5^c^	2.5^de^	2.5^def^	0.5^ cd^	5.5^be^	22.9^a^	6.0^bf^	< 0.001
	13.4^a^	11.9^a^	2.5^b^	3.0^bd^	10.9^ae^	1.0^b^	11.4^ae^	20.9^c^	6.5^de^	< 0.001
	7.5^a^	4.0^ab^	0.0	1.5^b^	4.0^ab^	0.0	4.0^ab^	20.9^c^	3.5^b^	< 0.001
	1.0	0.5	0.5	0.5	0.5	0.0	1.0	2.5	1.0	0.209
	16.4^af^	14.9^acf^	2.5^b^	5.5^bd^	10.0^ cd^	0.5^e^	10.9^acg^	19.4^f^	9.0^dg^	< 0.001
	19.4^a^	14.9^ac^	2.0^b^	3.5^b^	12.9^cde^	2.5^b^	12.9^ce^	19.4^ad^	8.0^e^	< 0.001
	7.0^ac^	12.9^b^	9.0^abc^	10.9^ab^	6.0^ac^	4.5^c^	6.0^ac^	6.5^ac^	8.0^abc^	0.019
	2.0^a^	2.0^a^	3.5^a^	4.0^a^	4.0^a^	2.5^a^	9.0^b^	2.5^a^	4.5^a^	0.002
	15.4^a^	16.9^a^	0.0	0.5^b^	9.0^c^	0.0	8.0^c^	23.4^d^	9.0^c^	< 0.001
	0.5	1.5	0.0	1.5	0.5	0.0	0.5	0.5	1.5	0.375
	13.9^a^	8.0^b^	0.0	1.0^ce^	7.5^bf^	0.5^c^	5.5^bf^	24.9^d^	3.5^ef^	< 0.001
	10.0^ad^	6.5^ace^	0.5^b^	1.5^bc^	6.0^ace^	0.0	4.0^ce^	11.4^d^	4.5^e^	< 0.001
	6.0	6.0	6.5	6.0	7.5	10.4	5.5	5.0	8.0	0.289
	5.5^a^	5.0^a^	6.0^a^	7.0^ab^	6.0^a^	5.5^a^	11.4^b^	3.5^a^	7.0^a^	0.025
Neutral valence										
	21.4^ad^	24.4^ade^	23.4^ade^	35.3^bf^	25.9^ae^	10.9^c^	24.9^ade^	18.9^d^	30.3^ef^	< 0.001
	5.5^a^	5.5^a^	14.4^b^	12.9^bc^	13.4^bc^	7.5^ac^	16.4^b^	5.0^a^	11.9^bc^	< 0.001
	2.0	3.5	2.5	4.0	4.5	1.0	4.5	5.0	4.0	0.255

C1—crowding, C2—anterior open bite, C3—interincisal diastema, C4—increased overjet and deep bite (Class II division 1), C5—anterior crossbite (Class III), C6—ideal occlusion, C7—unilateral posterior crossbite, C8—anterior open bite plus bilateral posterior crossbite plus crowding, C9—deep bite (Class II division 2)

^a,^^b,c,d,e,f,g^ Different superscript letters indicate difference between conditions in the same row

^†^The conditions for comparison between the groups using the McNemar test were not met

**Fig. 3:**
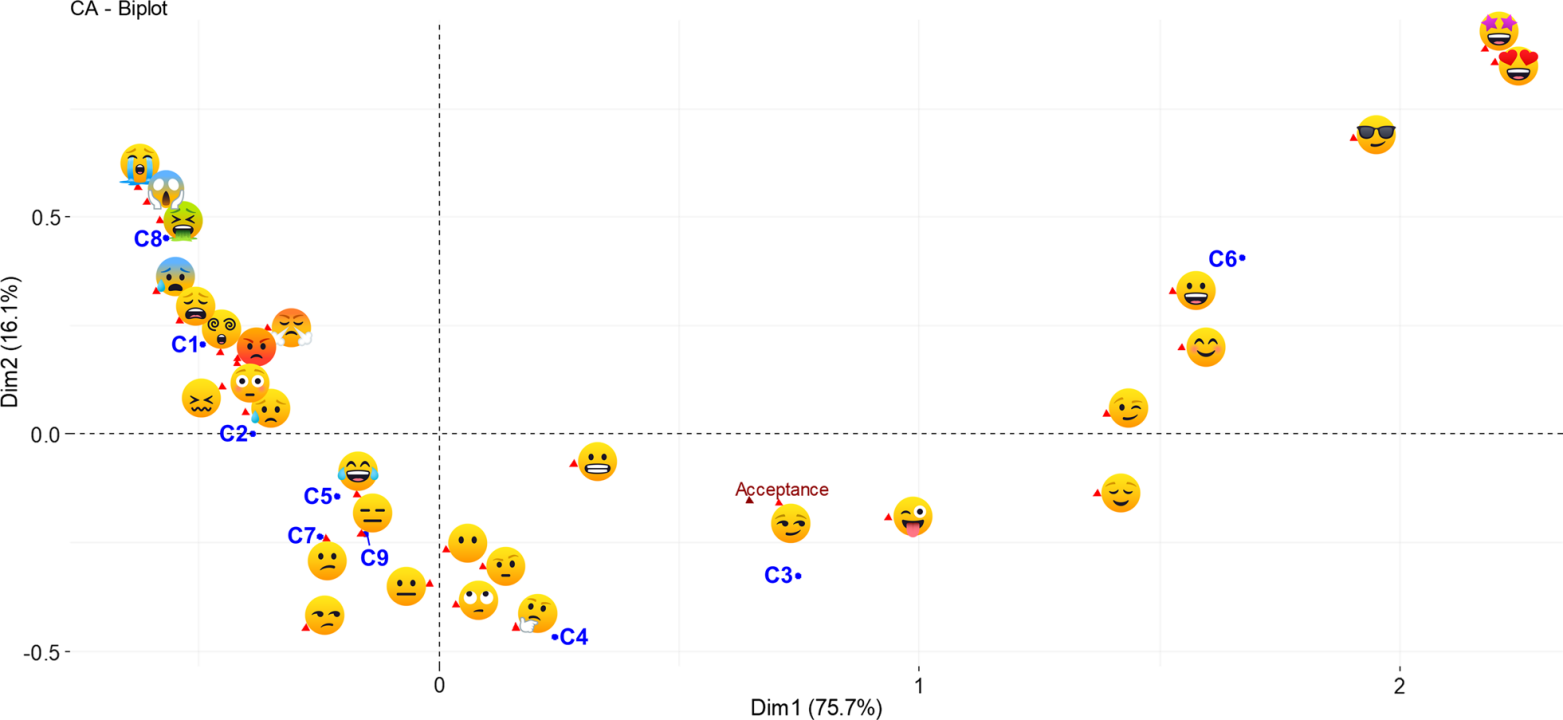
Correspondence analysis symmetric plot. C1—crowding, C2—anterior open bite, C3—interincisal diastema, C4—increased overjet and deep bite (Class II division 1), C5—anterior crossbite (Class III), C6—ideal occlusion, C7—unilateral posterior crossbite, C8—anterior open bite plus bilateral posterior crossbite plus crowding, and C9—deep bite (Class II division 2)

Correspondence analysis showed a significant dependence between the occlusal conditions and the frequency of choice of *emoji* (chi-square: 2237.813, *P* > 0.001). The data set of these variables was summarized in dimensions 1 (horizontal axis of the factorial map) and 2 (vertical axis) that approximately explained 75.7% and 16.1%% of the inertia respectively. This corresponds to a cumulative 91.8% of the total inertia retained by these two dimensions. Dimension 1 was related to the emotional valence of *emoji*. Conditions C6 (63.4%) and C3 (10.9%) at the positive pole of the horizontal axis, and C8 (10.8%) at the negative pole, as well as *emoji*  (15.8%),  (12.8%), and  (12, 1%), were the ones that most contributed to the definition of dimension 1. This axis was mainly characterized by the opposition of condition C6 and *emoji*  in the positive pole; and, C8 and various emoji with small contributions (*i.e.*,  ) in the negative pole. On the other hand, dimension 2 was related the emotional arousal/activation of *emoji*. Conditions C8 (32.1%) and C6 (17.7%) at the positive pole of the vertical axis, and C4 (20.4%) at the negative pole, as well as *emoji*  (14.3%),  (10.3%), and  (10.2%) were the ones that most contributed to the definition of dimension 2. This axis was characterized mainly by the opposition of conditions C6, C8, and *emoji*  , in the positive pole; and conditions C3, C4, and *emoji*  in the negative pole. Figure 3 shows the global pattern of the data in a symmetric plot. Conditions and *emoji* located on the right side of the factor map were associated with higher overall acceptance (supplementary variable, dimension 1 cos2: 0.922; dimension 2 cos2: 0.053).

The frequency of choice of  for condition C6 was significantly higher compared to the other conditions. Similarly,  evidenced a higher frequency for C3;  presented a higher frequency for C8;  showed a higher frequency for C1 and C2; and  , the highest frequency of choice for conditions C5, C7, and C9. Additional file 2: Fig. S2 shows the relationship between oral conditions and *emoji* in an asymmetric plot.

**Fig. 4:**
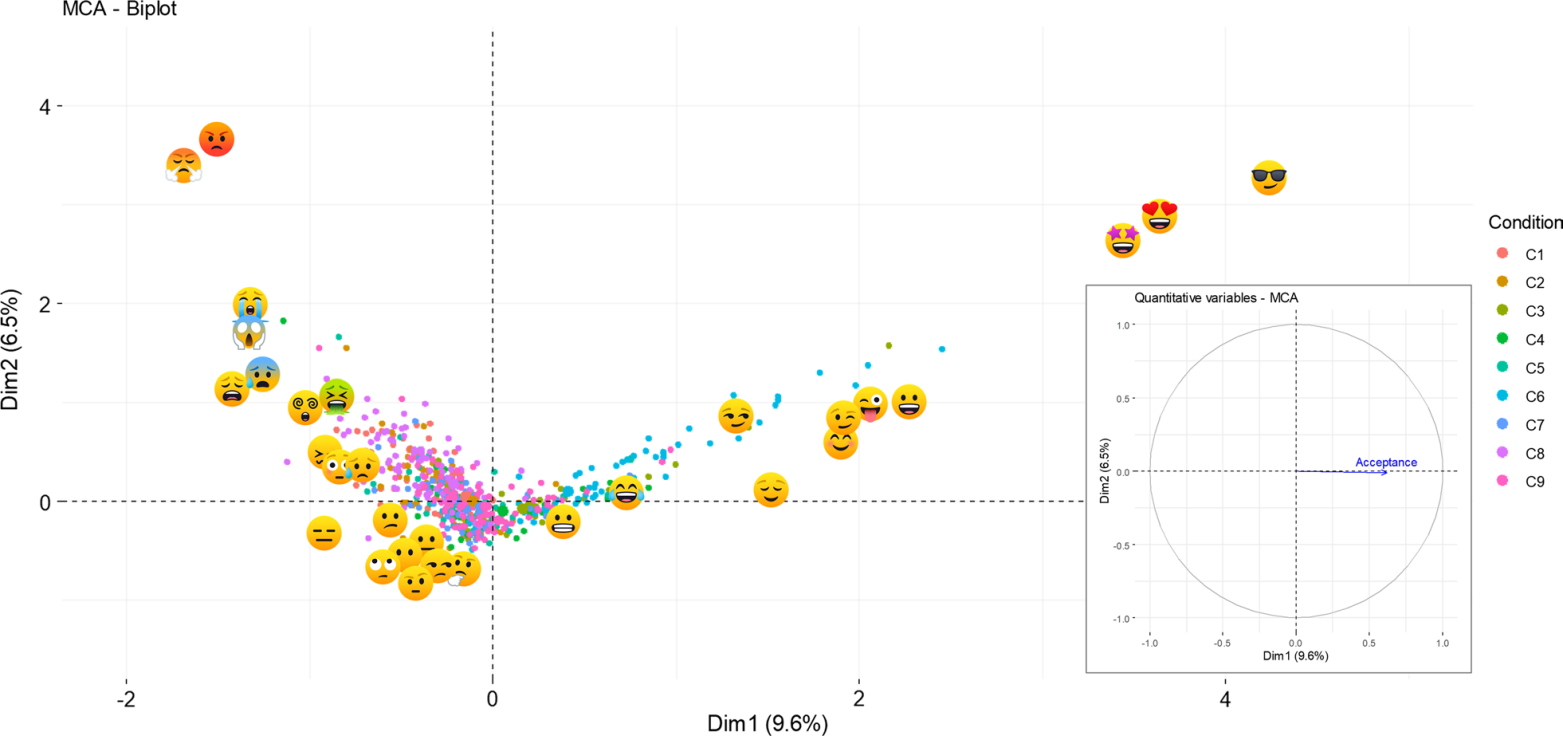
Multiple correspondence analysis factor map. C1—crowding, C2—anterior open bite, C3—interincisal diastema, C4—increased overjet and deep bite (Class II division 1), C5—anterior crossbite (Class III), C6—ideal occlusion, C7—unilateral posterior crossbite, C8—anterior open bite plus bilateral posterior crossbite plus crowding, and C9—deep bite (Class II division 2)

Multiple correspondence analysis evidenced that individual responses for the most rejected conditions were grouped on the left side of the map while responses for the most accepted conditions were on the right side (Fig. 4). Conditions and *emoji* located on the right side were associated with higher overall acceptance scores. Besides, although the first two axes only represented 16.1% of the total inertia, a similar *emoji* disposition to that of the simple correspondence analysis could be observed for these dimensions (Fig. 4). The horizontal axis (dimension 1) was characterized by the opposition of *emoji*  in the positive pole; and,  in the negative pole. On the other hand, the vertical axis (dimension 2) showed *emoji*  with higher contributions on the positive pole; and, several *emoji* with low contributions on the negative pole (*i.e.*,  ).

Only weak significant correlations were identified between overall acceptance scores and *emoji* frequency counts (*P* < 0.05; Table 3). Positive correlations between acceptance and  were observed. On the other hand,  were negatively correlated to acceptance scores.

**Table 3:** Correlations between mean overall acceptance and *emoji* frequency counts

*Emoji*	Kendall’s Tau-b	*P*-value
Positive valence		
	0.138	0.014*
	0.073	0.213
	0.019	0.740
	0.111	0.048*
	0.130	0.025*
	0.102	0.081
	0.064	0.266
	0.207	< 0.001*
	0.135	0.021*
	0.148	0.009*
Negative valence		
	− 0.127	0.015*
	− 0.118	0.024*
	− 0.033	0.572
	− 0.056	0.314
	− 0.036	0.515
	− 0.101	0.073
	− 0.116	0.046*
	− 0.012	0.825
	0.047	0.389
	0.061	0.270
	0.030	0.595
	− 0.075	0.175
	0.050	0.390
	− 0.105	0.061
	0.003	0.963
	0.129	0.021*
	0.049	0.380
Neutral valence		
	− 0.021	0.686
	0.126	0.021*
	0.027	0.638

* indicates significant correlation

## Discussion

The use of *emoji* in the field of health is not entirely new [8]. *Emoji* have already been used, for example, as auxiliary tools in identification of mental illnesses such as depression [24], development of strategies to guide behaviors related to health [25], or monitoring moods during care [26]. The development of strategies to evaluate emotions can facilitate the establishment of a better connection between the professional and patients, identifying negative emotions that require a multidisciplinary approach and positive ones that require maintenance and/or reinforcement. There is a gap in knowledge about the emotions generated by stimuli related to oral conditions or diseases. Our findings show for the first time that *emoji* have adequate discriminative ability and that these tools would allow determining emotional profiles in the face of specific oral conditions.

Previous studies in the research field of consumers' food preferences have already demonstrated the ability of these tools to discriminate tasted samples of different product categories [9, 11, 27, 28]. The results of the present study confirmed that *emoji* have discriminatory ability when judging preferences. From a general perspective, the majority of positive *emoji* differentiate more clearly between conditions with greater acceptance on one hand (*i.e.*, C6 and C3) and the rest of the malocclusions; and, most negative *emoji* between conditions with greater rejection (*i.e.*, C1, C2, and C8) and the others. It is important to mention that when conditions with similar overall acceptance are considered (*e.g.*, C1, C2, and C8), the differences in the frequencies of use for each *emoji* are smaller, and in many cases not significant. This pattern of results was previously evidenced: *emoji* would not have the same ability to discriminate between samples with similar liking [11]. It is important to mention that there was a limitation that most of the malocclusions were severe, with the exception of C3 and C6 ("ideal occlusion"). This could have caused the difficulty of discriminating between conditions with low acceptance. Despite this, it must be emphasized that the very severe conditions (*i.e.*, C8) were clearly differentiated from the other malocclusions.

It has been suggested that self-reported measures of emotional response are better captured along dimensions rather than specific emotional states (*e.g.*, anger, sadness, fear) [1]. Positive *emoji* were located on the right side of the graph (*i.e.*,  ), negative *emoji* on the left side (*i.e.*,  ), and more neutral emoji near the origin of the factor map (*i.e.*,  ), demonstrating the contribution of the *emoji* valence in the definition of dimension 1. In accordance with this, previous studies that evaluated food samples demonstrated a similar arrangement of the study variables using word-based methods and *emoji* [9, 11, 29–32]. On the other hand, and in accordance with the previously reported [11], dimension 2 was related to the emotional arousal/activation. *Emoji* with high emotional arousal were on the positive pole of the map (*i.e.*,  ) and *emoji* with low emotional arousal on the negative pole (*i.e.*,  ). Based on what was observed in the asymmetric plot of the correspondence analysis, and from a general perspective, it can be said that conditions with less acceptance (*i.e.*, C1, C2, C5, C8, C7, and C9) were associated with negative emotions, and conditions with better acceptance (*i.e.*, C3, C4, and C6) were associated with positive emotions, both with variation in emotional arousal/activation.

Regarding the multiple correspondence analysis, the pattern of individual responses using *emoji* for the different conditions was similarly based on emotional valence and arousal/activation. Most of the responses for the conditions with the highest rejection were located on the left side, while responses for the conditions with the highest acceptance were located on the right side of the factorial map. Larsen and Diener's circumplex two-dimensional model organize emotions into quadrants as follows: unpleasant/high activation (upper left quadrant, 45°), pleasant/high activation (upper right quadrant, 135°), pleasant/low activation (lower right quadrant, 225°) and unpleasant/low activation (lower left quadrant, 315°) [33]. As previously reported [11], it was evidenced that *emoji* were distributed at angles close to 45° and 135° in the first and second quadrants, respectively. According to the arrangement of *emoji*, it can be said that  would be more pleasant and activated than  ; while  would be more unpleasant and activated than  . We emphasize that this disposition must be confirmed by additional investigations since both dimensions explained a low percentage of total inertia.

Supporting all the mentioned results, both correspondence analyses demonstrated that higher overall acceptance scores were related to conditions and *emoji* located on the right side of the factorial maps. Correlations were additionally evaluated in a complementary way to interpret the arrangement of *emoji* in the factor maps of the correspondence analysis. These calculations were carried out with the premise that positive emotions would correspond to greater acceptance and, conversely, negative emotions to less acceptance. Our findings confirmed this hypothesis. Although the strength of the correlations was weak, a trend could be observed in these results: positive *emoji* were positively correlated, and negative *emoji* negatively correlated with individuals' acceptance. It should be mentioned that a weak correlation that is statistically significant suggests that, in fact, both variables are correlated but that there were other important determinants as well. We speculate that because various *emoji* would have similar behavior (clustering observed in correspondence analysis factor maps), it is likely that participants would have interpreted and used different *emoji* to express the same emotional response. Consequently, this would have caused frequency of use to be distributed across different *emoji*, resulting in several *emoji* with weak significant correlations rather than a few *emoji* showing strong significant correlations with acceptance scores.

Interestingly, emoji  , classified a priori as negative and neutral, respectively, showed a positive correlation with the acceptance scores. It has been proven that *emoji* are prone to generating multiple interpretations due to the complexity of the gestures they represent [19, 34, 35]. It is probable that neutral *emoji* located close to the origin of the factorial map have depicted different meanings for individuals.  (“grimacing face”), as indicated in *Emojipedia*, can represent negative or tense emotions, specifically nervousness, embarrassment, or awkwardness; however, individuals could have interpreted it as a positive low arousal emotion gesture. Similarly, the tone of  (“thinking face”) can be highly variable (*i.e.*, serious, playful, puzzled, skeptical, and mocking [based on *Emojipedia*]). Regarding these findings, we must mention that pre-classification of *emoji* according to their emotional valence was based on previous studies that did not evaluate oral conditions; therefore, it was even expected that some *emoji* would acquire different interpretations in this new context. *Emoji* which showed contradictory results possibly reflecting variations in interpretation by individuals, would not be suitable candidates to be used in subsequent phases of research. However, additional investigations should be carried out on how people interpret the *emoji* meaning related to other oral conditions different from malocclusions.

Currently, in dental research, the emotional component is usually evaluated as a domain within a broader construct that is quality of life. The measurement instruments contain Likert scales as response options to judge how often a certain negative emotion has been experienced in a recent period [36]. Another common approach is the use of subjective scales to obtain information with emotional content. The visual analog scale, ordinal rating scale, or similar, are frequently used to measure pleasantness in the face of different dentofacial conditions [37, 38]. Both approaches only reflect a partial picture of what the emotional response is since they are focused on specific emotional states (*e.g.*, sadness, shame, pleasant). As mentioned above, it has been suggested that self-reported measures of emotional response are better captured along dimensions rather than specific states [1]. Although verbal methods would also meet this premise [29–32], these do not capture automatic emotional evoked associations [3]. On the contrary, *emoji*, being images that reflect expressions commonly used in people's interactions [6], would have the ability to intuitively provide this information [6, 7]. In this sense, our results demonstrate that *emoji* are promising alternatives for use in measuring emotional response in the face of oral conditions since these tools would have the ability of providing information on the emotional profile of individuals as such (*i.e.*, a positive, neutral or negative response). In addition, considering that *emoji* manifest different emotional arousal/activation, these tools would possibly allow graded the intensity of this response. We want to emphasize that based on the current methodology and results, it is not possible to recommend the use of *emoji* over any other consolidated method to measure emotional response. It is true that this is our ultimate goal; however, the reported findings are only a necessary preliminary step towards that goal. The main purpose of this research was to evaluate some psychometric properties of *emoji* and determine if these tools are adequate means of response for the construction of an instrument that allows us to measure the emotional response in the field of dentistry. Results of the different implemented analyses must be evaluated together to select the best *emoji*. In a summary, and based on the frequency of use, discriminating ability, relationship with the conditions of greater and lesser liking, and observed correlation patterns, the following *emoji* could be recommended for next step research (ordered as follows, positives with high arousal → positives with low arousal → neutral → negative with low arousal → negative with high arousal):  .

It should be mentioned that due to exploratory nature of this research, it has some limitations that must be considered when evaluating the reported results. First, no sample size calculation was performed. Due to the lack of prior evidence on the matter, we opted to work with a convenience sample; therefore, there is a possibility that our results are not powerful enough. An important point to keep in mind is that the present evaluations were issued on images of occlusal conditions in an edited smile context. Variations in the shape of the lips, smile design, gingival exposure, and of course, the facial expression in the case of complete face evaluations, could generate different judgments against these conditions. Furthermore, only one image was presented to characterize each of the conditions studied. We consider it appropriate to interpret the present findings as related to 'high' or 'low' acceptance occlusal conditions, instead of relating them to specific malocclusions, since these conditions present great variability and even several of them can occur simultaneously in the clinical context. Another relevant point to consider is that the participants' judgments were issued towards conditions that they did not necessarily present. Regression analyses showed that the variables self-perception of the smile esthetics and/or bite, as well as the previous experience of orthodontic treatment could influence the selection of some *emoji* (  , respectively; Additional file 4: Table S2). It is likely that the emotional response would be different if the participants evaluated their own condition. In this sense, new research on the use of emoji should be carried out from this perspective. Finally, our results also evidenced that *emoji* choice (for  ; Additional file 4: Table S2) could also be modified by the age and/or sex of the individuals. A previous study demonstrated that there are age-related differences in the use of *emoji* [39]. Given that the present investigation was carried out in a convenience sample that included only adults and was predominantly female, the present results are not generalizable to other age groups such as children.

## Conclusion

Based on our results, we conclude that *emoji* are promising tools to be incorporated into an instrument that measures emotional response of adults in orthodontics, due to their adequate discriminating ability and the fact that these tools would allow determining emotional profiles against specific occlusal conditions.

## Supplementary Information

**Additional file 1. Fig. S1.** Frequency of indication as the most accepted and most rejected condition. C1—crowding, C2—anterior open bite, C3—interincisal diastema, C4—increased overjet and deep bite (Class II division 1), C5—anterior crossbite (Class III), C6—ideal occlusion, C7—unilateral posterior crossbite, C8—anterior open bite plus bilateral posterior crossbite plus crowding, and C9—deep bite (Class II division 2).

**Additional file 2. Fig. S2.** Correspondence analysis asymmetric plot. C1—crowding, C2—anterior open bite, C3—interincisal diastema, C4—increased overjet and deep bite (Class II division 1), C5—anterior crossbite (Class III), C6—ideal occlusion, C7—unilateral posterior crossbite, C8—anterior open bite plus bilateral posterior crossbite plus crowding, and C9—deep bite (Class II division 2).

**Additional file 3. Table S1.** *Emoji* meanings (http://emojipedia.org/).

**Additional file 4. Table S2.** Contribution of explanatory variables in the choice of *emoji* within the adjusted logistic regression model.

## Data Availability

The datasets generated during and/or analyzed during the current study are available from the corresponding author on reasonable request.

## Abbreviations

IOTN: Index of orthodontic treatment need
EC: Esthetic component
CI: Confidence interval
