# Supplementary figures and images for: Emoji as promising tools for emotional evaluation in orthodontics
Source: Prog Orthod. 2022 Jul 18;23:28. doi: 10.1186/s40510-022-00418-3 (PMC9288943; doi:10.1186/s40510-022-00418-3)

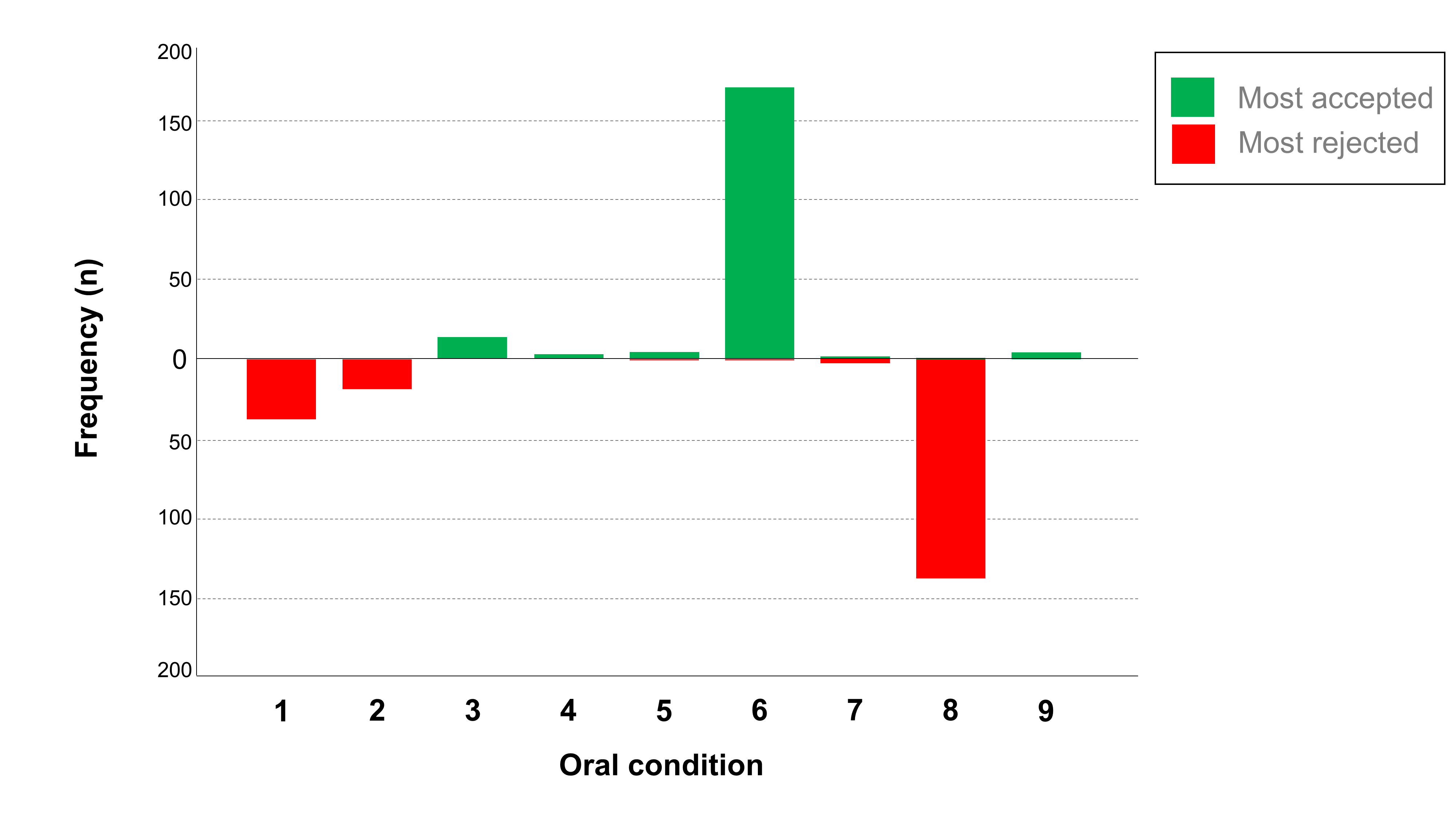

Supplement: Supplementary file 1 — Additional file 1. Fig. S1. Frequency of indication as the most accepted and most rejected condition. C1—crowding, C2—anterior open bite, C3—interincisal diastema, C4—increased overjet and deep bite (Class II division 1), C5—anterior crossbite (Class III), C6—ideal occlusion, C7—unilateral posterior crossbite, C8—anterior open bite plus bilateral posterior crossbite plus crowding, and C9—deep bite (Class II division 2). [file 40510_2022_418_MOESM1_ESM.tif]

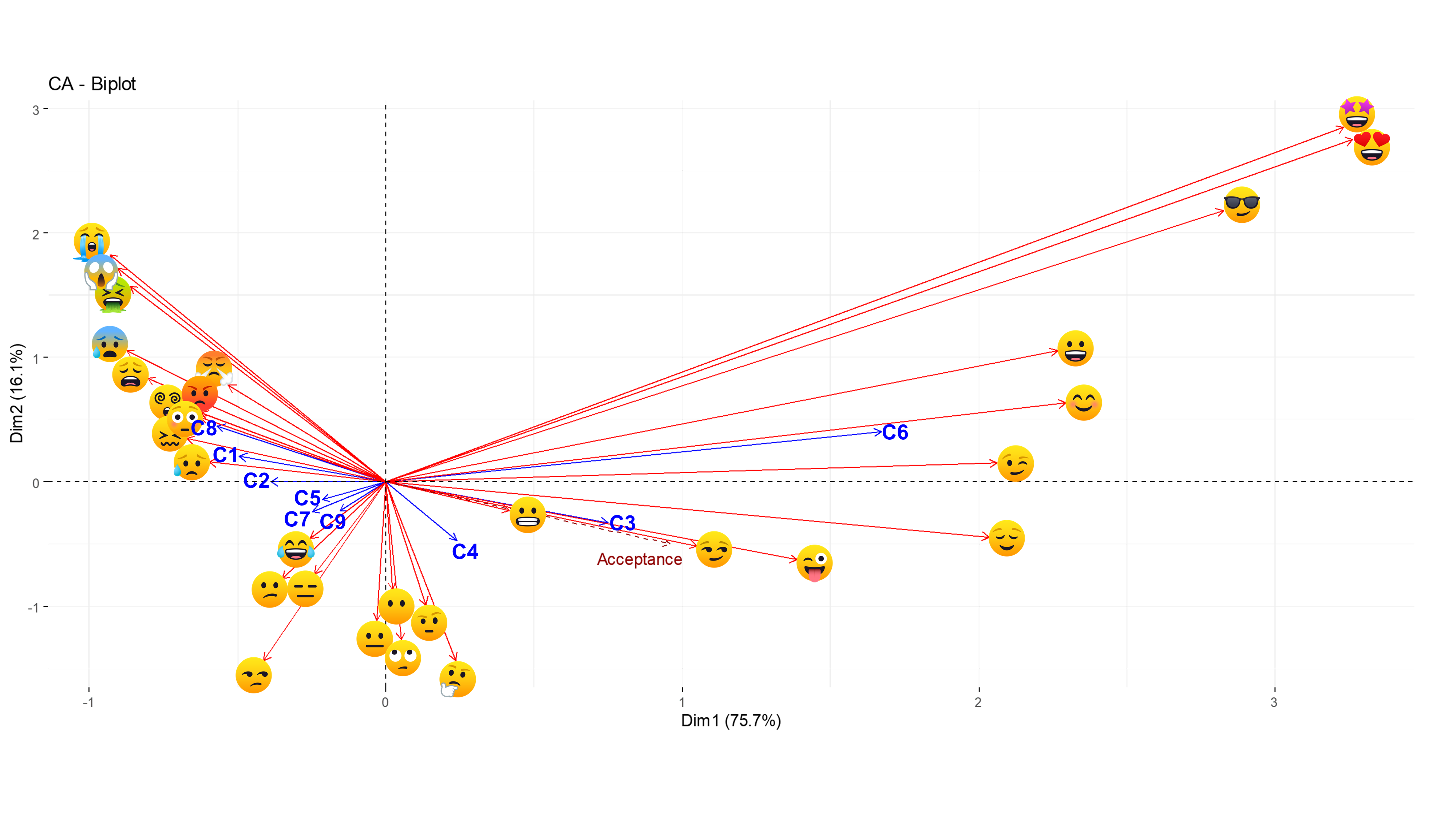

Supplement: Supplementary file 2 — Additional file 2. Fig. S2. Correspondence analysis asymmetric plot. C1—crowding, C2—anterior open bite, C3—interincisal diastema, C4—increased overjet and deep bite (Class II division 1), C5—anterior crossbite (Class III), C6—ideal occlusion, C7—unilateral posterior crossbite, C8—anterior open bite plus bilateral posterior crossbite plus crowding, and C9—deep bite (Class II division 2). [file 40510_2022_418_MOESM2_ESM.tif]
