# Supplementary material for: Emoji as promising tools for emotional evaluation in orthodontics
Source: Prog Orthod. 2022 Jul 18;23:28. doi: 10.1186/s40510-022-00418-3 (PMC9288943; doi:10.1186/s40510-022-00418-3)
Supplement: Supplementary file 3 — Additional file 3. Table S1. Emoji meanings (http://emojipedia.org/). [file 40510_2022_418_MOESM3_ESM.docx]

Supplementary Table 1. *Emoji* meanings (<http://emojipedia.org/>).

| *Emoji* | *Emojipedia* name | *Emojipedia* meaning |
| --- | --- | --- |
| 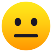 | Neutral Face | Intended to depict a neutral sentiment but often used to convey mild irritation and concern or a deadpan sense of humor. |
| 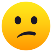 | Confused Face | While it can convey confusion, it is commonly used for moderate sadness, disappointment, and frustration, thanks to its frown. |
| 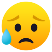 | Sad but Relieved Face | Commonly conveys mild degrees of frustration and sadness. |
| 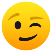 | Winking Face | May signal a joke, flirtation, hidden meaning, or general positivity. Tone varies, including playful, affectionate, suggestive, or ironic. |
| 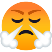 | Face with Steam From Nose | May convey various negative emotions, including irritation, anger, and contempt. May also convey feelings of pride, dominance, and empowerment. |
| 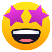 | Star-Struck | May express that someone or something is amazing, fascinating, impressive, or exciting. |
| 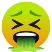 | Face Vomiting | May represent physical illness or disgust, more intensely so than 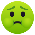 Nauseated Face. |
| 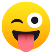 | Winking Face with Tongue | Often conveys a sense of fun, excitement, wackiness, buffoonery, or joking. |
| 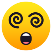 | Dizzy Face | May convey a heightened or hyperbolic sense of such feelings as shock, surprise, disbelief, awe, and amazement, as if staggered to the point of disorientation (*i.e.*, dizzy). May also represent sickness, nausea, intoxication, and death, *e.g.*, slang *I’m dead*! |
| 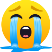 | Loudly Crying Face | May convey inconsolable grief but also other intense feelings, such as uncontrollable laughter, pride or overwhelming joy. |
| 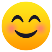 | Smiling Face with Smiling Eyes | Often expresses genuine happiness and warm, positive feelings. |
| 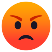 | Pouting Face | Bears the same expression as 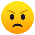 Angry Face on most platforms and may convey more intense degrees of anger, *e.g.*, hate or rage. |
| 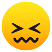 | Confounded Face | May be used to represent being overcome with various emotions, including irritation, frustration, disgust, and sadness, as if to the point of defeat. |
| 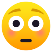 | Flushed Face | Intended to depict such feelings as embarrassment, but meaning very widely varies. Other senses include flattery, surprise, disbelief, admiration, affection, and excitement. |
| 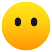 | Face Without Mouth | Meaning widely varies, but commonly conveys speechlessness, humility, and silence. May also convey moderately negative emotions, such as disappointment, frustration, or sadness. |
| 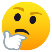 | Thinking Face | Often used to question or scorn something or someone, as if saying *Hmm, I don't know about that*. Tone varies, including earnest, playful, puzzled, skeptical, and mocking. |
| 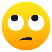 | Face with Rolling Eyes | As with the gesture of an eye-roll, commonly conveys moderate disdain, disapproval, frustration, or boredom. Tone varies, including playful, sassy, resentful, and sarcastic, as if saying Yeah, whatever. |
| 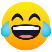 | Face with Tears of Joy | Widely used to show something is funny or pleasing. |
| 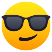 | Smiling Face with Sunglasses | Often used to convey the slang sense of cool. May also express a confident, carefree attitude or that something is excellent. |
| 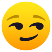 | Smirking Face | Often used to convey flirtation or sexual innuendo. |
| 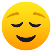 | Relieved Face | Conveys various pleasant feelings, including contentment, calm, peace, and relief. May also convey feelings of happiness or good-natured humor more generally. |
| 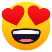 | Smiling Face with Heart-Eyes | Often conveys enthusiastic feelings of love, infatuation, and adoration, *e.g.*, *I love/am in love with this person or thing.* |
| 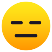 | Expressionless Face | May convey a sense of frustration or annoyance more intense than suggested by 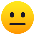 Neutral Face, as if taking a moment to collect itself. |
| 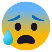 | Anxious Face with Sweat | Meaning widely varies, but commonly conveys such feelings as sadness, disappointment, fear, and anxiety. Similar to 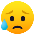 Sad but Relieved Face, but with a blue head and larger frown. |
| 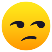 | Unamused Face | May convey a variety of negative emotions, including irritation, displeasure, grumpiness, and skepticism, as if giving the side-eye. |
| 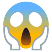 | Face Screaming in Fear | While intended to represent horror and fright, it commonly conveys such feelings as shock, awe, disbelief, and intense excitement, as a screaming fan. |
| 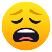 | Weary Face | May convey various feelings of frustration, sadness, amusement, and affection. Often playful in tone. |
| 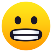 | Grimacing Face | May represent a range of negative or tense emotions, especially nervousness, embarrassment, or awkwardness (*e.g.*, *Eek!*). |
| 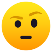 | Face with Raised Eyebrow | Conveys a wide variety of sentiments, including suspicion, skepticism, concern, consideration, disbelief, and disapproval. |
| 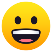 | Grinning Face | Often conveys general pleasure and good cheer or humor. |
