# Supplementary material for: Emoji as promising tools for emotional evaluation in orthodontics
Source: Prog Orthod. 2022 Jul 18;23:28. doi: 10.1186/s40510-022-00418-3 (PMC9288943; doi:10.1186/s40510-022-00418-3)
Supplement: Supplementary file 4 — Additional file 4. Table S2. Contribution of explanatory variables in the choice of emoji within the adjusted logistic regression model. [file 40510_2022_418_MOESM4_ESM.docx]

Supplementary Table 2. Contribution of explanatory variables in the choice of *emoji* within the adjusted logistic regression model.

| *Emoji* | Oral condition | R^2^ | Explanatory variables *P*-value | | | | | | | | | | | | | | |
| --- | --- | --- | --- | --- | --- | --- | --- | --- | --- | --- | --- | --- | --- | --- | --- | --- | --- |
|  |  |  | Age | Sex (Male†-Female) | Self-perception of smile esthetics (Good†) | |  | Self-perception of bite (Good†) | |  | Orthodontic treatment experience (Non treated†) | |  | Daily frequency of use of *emoji* (Never†) | | | |
|  |  |  |  |  | Moderate | Bad |  | Moderate | Bad |  | Treated | In treatment |  | Rarely | Some times | Frequently | Always |
| Positive valence | | | | | | | | | | | | | | | | | |
| 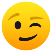 | C3 | 0.087 | 0.539 | 0.366 | 0.824 | 0.990 |  | 0.786 | 0.784 |  | 0.150 | 0.426 |  | 0.997 | 0.997 | 0.997 | 0.997 |
|  | C6 | 0.125 | 0.323 | 0.496 | 0.709 | 0.117 |  | 0.324 | 0.096 |  | 0.144 | 0.320 |  | 0.987 | 0.987 | 0.987 | 0.986 |
| 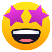 | C6 | 0.069 | 0.243 | 0.717 | 0.501 | 0.443 |  | 0.703 | 0.523 |  | 0.264 | 0.450 |  | 0.992 | 0.992 | 0.992 | 0.992 |
| 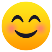 | C3 | 0.054 | 0.986 | 0.257 | 0.112 | 0.553 |  | 0.761 | 0.489 |  | 0.810 | 0.605 |  | 0.993 | 0.993 | 0.993 | 0.993 |
|  | C6 | 0.107 | 0.623 | 0.915 | 0.660 | 0.415 |  | 0.510 | 0.738 |  | 0.292 | 0.387 |  | 0.993 | 0.996 | 0.996 | 0.996 |
| 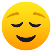 | C3 | 0.161 | 0.931 | 0.774 | 0.180 | 0.212 |  | 0.350 | 0.964 |  | 0.060 | 0.990 |  | 0.998 | >0.999 | >0.999 | >0.999 |
|  | C6 | 0.088 | 0.411 | 0.551 | 0.300 | 0.284 |  | 0.306 | 0.105 |  | 0.867 | 0.884 |  | 0.990 | 0.989 | 0.988 | 0.989 |
| 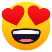 | C6 | 0.114 | 0.181 | 0.882 | 0.591 | 0.473 |  | 0.256 | 0.843 |  | 0.261 | 0.559 |  | >0.999 | 0.999 | 0.999 | 0.999 |
| 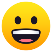 | C3 | 0.143 | 0.495 | 0.305 | 0.788 | 0.994 |  | 0.187 | 0.956 |  | 0.695 | 0.991 |  | >0.999 | >0.999 | >0.999 | >0.999 |
|  | C6 | 0.069 | 0.728 | 0.599 | 0.541 | 0.624 |  | 0.666 | 0.306 |  | **0.035*** | 0.263 |  | 0.989 | 0.988 | 0.988 | 0.988 |
| Negative valence | | | | | | | | | | | | | | | | | |
| 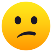 | C1 | 0.110 | 0.278 | 0.278 | 0.084 | **0.032*** |  | 0.676 | 0.716 |  | 0.154 | 0.427 |  | 0.985 | 0.985 | 0.986 | 0.985 |
|  | C2 | 0.104 | 0.411 | 0.236 | 0.589 | 0.720 |  | 0.473 | 0.557 |  | 0.247 | 0.515 |  | 0.986 | 0.987 | 0.986 | 0.986 |
|  | C3 | 0.128 | 0.147 | 0.365 | 0.103 | **0.048*** |  | 0.087 | 0.167 |  | 0.149 | 0.555 |  | 0.984 | 0.984 | 0.984 | 0.984 |
|  | C4 | 0.057 | 0.426 | **0.046*** | 0.258 | 0.551 |  | 0.309 | 0.670 |  | 0.938 | 0.703 |  | 0.989 | 0.989 | 0.989 | 0.989 |
|  | C5 | 0.101 | 0.616 | 0.203 | 0.883 | 0.449 |  | 0.197 | 0.650 |  | 0.887 | 0.641 |  | 0.989 | 0.989 | 0.988 | 0.988 |
|  | C7 | 0.067 | 0.914 | 0.686 | 0.657 | 0.339 |  | 0.537 | 0.636 |  | **0.042*** | 0.079 |  | 0.985 | 0.985 | 0.985 | 0.985 |
|  | C8 | 0.042 | 0.218 | 0.767 | 0.885 | 0.811 |  | 0.649 | 0.625 |  | 0.183 | 0.257 |  | 0.988 | 0.988 | 0.988 | 0.988 |
|  | C9 | 0.078 | 0.260 | 0.364 | 0.166 | 0.248 |  | 0.642 | 0.528 |  | 0.081 | 0.298 |  | 0.986 | 0.986 | 0.986 | 0.986 |
| 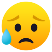 | C1 | 0.154 | **0.015*** | 0.901 | **0.015*** | 0.950 |  | 0.629 | 0.896 |  | 0.239 | **0.010*** |  | 0.987 | 0.986 | 0.986 | 0.987 |
|  | C2 | 0.043 | 0.428 | 0.860 | 0.089 | 0.524 |  | 0.607 | 0.310 |  | 0.475 | 0.590 |  | 0.986 | 0.987 | 0.987 | 0.987 |
|  | C4 | 0.120 | 0.600 | 0.214 | 0.171 | 0.217 |  | 0.911 | 0.092 |  | 0.350 | 0.580 |  | >0.999 | 0.997 | 0.997 | 0.997 |
|  | C5 | 0.160 | 0.297 | 0.074 | 0.405 | 0.989 |  | 0.197 | 0.147 |  | 0.679 | 0.465 |  | 0.996 | 0.996 | 0.996 | 0.996 |
|  | C7 | 0.133 | 0.406 | 0.365 | 0.059 | 0.094 |  | 0.441 | **0.015*** |  | 0.110 | 0.275 |  | 0.986 | 0.985 | 0.986 | 0.986 |
|  | C8 | 0.126 | 0.122 | 0.179 | 0.780 | 0.742 |  | 0.163 | 0.366 |  | 0.963 | 0.302 |  | 0.986 | 0.986 | 0.987 | 0.986 |
|  | C9 | 0.118 | 0.573 | 0.256 | 0.051 | 0.441 |  | 0.426 | 0.062 |  | 0.572 | 0.077 |  | 0.986 | 0.987 | 0.987 | 0.987 |
| 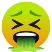 | C1 | 0.095 | 0.629 | 0.092 | 0.165 | 0.313 |  | 0.473 | 0.098 |  | 0.070 | 0.452 |  | 0.989 | 0.989 | 0.989 | 0.989 |
|  | C8 | 0.071 | 0.664 | **0.023*** | 0.490 | 0.726 |  | 0.665 | 0.689 |  | 0.834 | 0.241 |  | 0.987 | 0.988 | 0.988 | 0.988 |
| 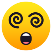 | C1 | 0.056 | 0.445 | 0.868 | 0.919 | 0.368 |  | 0.663 | 0.232 |  | 0.333 | 0.635 |  | 0.993 | 0.993 | 0.992 | 0.992 |
|  | C2 | 0.073 | 0.760 | 0.143 | 0.250 | 0.884 |  | 0.631 | 0.439 |  | 0.240 | 0.411 |  | 0.992 | 0.993 | 0.993 | 0.993 |
|  | C5 | 0.091 | 0.751 | 0.067 | 0.342 | 0.993 |  | 0.352 | 0.826 |  | 0.550 | 0.768 |  | >0.999 | 0.996 | 0.997 | 0.997 |
|  | C7 | 0.177 | **0.043*** | 0.316 | 0.468 | 0.488 |  | 0.540 | 0.755 |  | **0.014*** | 0.442 |  | >0.999 | 0.997 | 0.997 | 0.997 |
|  | C8 | 0.152 | 0.799 | **0.026*** | 0.460 | 0.434 |  | **0.039*** | 0.152 |  | 0.139 | 0.686 |  | 0.986 | 0.985 | 0.985 | 0.985 |
| 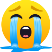 | C8 | 0.080 | 0.746 | 0.397 | 0.053 | 0.286 |  | 0.893 | 0.279 |  | 0.197 | 0.560 |  | 0.988 | 0.987 | 0.987 | 0.987 |
| 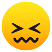 | C1 | 0.067 | 0.352 | 0.795 | 0.516 | 0.655 |  | 0.981 | 0.919 |  | 0.923 | 0.375 |  | 0.988 | 0.988 | 0.988 | 0.987 |
|  | C2 | 0.133 | **0.023*** | 0.973 | 0.700 | 0.331 |  | 0.737 | 0.051 |  | 0.770 | 0.636 |  | 0.993 | 0.993 | 0.992 | 0.993 |
|  | C5 | 0.113 | 0.756 | 0.318 | 0.258 | 0.429 |  | 0.100 | 0.317 |  | 0.396 | 0.423 |  | 0.993 | 0.993 | 0.993 | 0.993 |
|  | C7 | 0.090 | 0.109 | 0.349 | 0.808 | 0.732 |  | 0.890 | 0.372 |  | 0.209 | 0.378 |  | 0.992 | 0.993 | 0.993 | 0.993 |
|  | C8 | 0.141 | 0.352 | 0.922 | 0.750 | 0.307 |  | 0.054 | 0.174 |  | 0.900 | 0.470 |  | 0.993 | 0.996 | 0.996 | 0.996 |
| 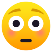 | C1 | 0.166 | 0.903 | 0.310 | 0.271 | 0.989 |  | 0.065 | **0.044*** |  | 0.107 | 0.093 |  | 0.997 | 0.997 | 0.997 | 0.997 |
|  | C2 | 0.075 | 0.411 | 0.541 | 0.877 | 0.990 |  | 0.949 | 0.394 |  | 0.393 | 0.171 |  | 0.997 | 0.997 | 0.997 | 0.997 |
|  | C5 | 0.104 | 0.327 | 0.155 | 0.848 | 0.994 |  | 0.770 | 0.865 |  | 0.254 | 0.501 |  | >0.999 | 0.998 | 0.998 | 0.998 |
|  | C7 | 0.123 | 0.807 | 0.188 | 0.799 | 0.990 |  | 0.940 | 0.567 |  | **0.038*** | 0.085 |  | 0.997 | 0.997 | 0.997 | 0.997 |
|  | C8 | 0.125 | 0.438 | **0.013*** | 0.116 | 0.246 |  | 0.704 | 0.553 |  | 0.143 | 0.346 |  | 0.986 | 0.986 | 0.986 | 0.985 |
| 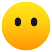 | C2 | 0.166 | 0.148 | **0.030*** | 0.863 | 0.112 |  | 0.675 | 0.340 |  | 0.549 | 0.059 |  | 0.992 | 0.992 | 0.992 | 0.992 |
|  | C4 | 0.118 | 0.488 | 0.123 | 0.233 | 0.697 |  | --- | --- |  | 0.098 | 0.052 |  | >0.999 | 0.997 | 0.997 | 0.997 |
| 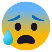 | C1 | 0.116 | 0.511 | 0.577 | 0.978 | 0.596 |  | 0.135 | 0.888 |  | 0.640 | 0.389 |  | >0.999 | 0.997 | 0.997 | 0.997 |
|  | C2 | 0.075 | 0.303 | 0.567 | 0.902 | 0.883 |  | 0.084 | 0.987 |  | 0.131 | 0.185 |  | 0.990 | 0.990 | 0.989 | 0.990 |
|  | C8 | 0.071 | 0.347 | 0.091 | 0.706 | 0.887 |  | 0.689 | 0.494 |  | 0.244 | 0.402 |  | 0.989 | 0.987 | 0.988 | 0.989 |
| 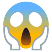 | C1 | 0.154 | 0.501 | 0.309 | 0.111 | 0.994 |  | 0.787 | 0.417 |  | 0.122 | 0.970 |  | >0.999 | 0.998 | 0.998 | 0.998 |
|  | C8 | 0.131 | 0.579 | 0.189 | **0.011*** | 0.097 |  | 0.998 | 0.065 |  | 0.186 | 0.064 |  | 0.989 | 0.988 | 0.988 | 0.989 |
| 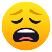 | C1 | 0.184 | 0.218 | 0.170 | 0.313 | 0.215 |  | 0.838 | 0.404 |  | 0.061 | 0.562 |  | >0.999 | 0.998 | 0.998 | 0.998 |
|  | C8 | 0.056 | 0.762 | 0.877 | 0.831 | 0.949 |  | --- | --- |  | 0.509 | 0.288 |  | >0.999 | 0.997 | 0.997 | 0.997 |
| 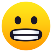 | C6 | 0.099 | 0.873 | 0.093 | 0.957 | 0.956 |  | 0.304 | 0.585 |  | 0.456 | 0.721 |  | 0.993 | 0.993 | 0.993 | 0.993 |
| 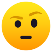 | C7 | 0.123 | 0.213 | 0.253 | 0.158 | 0.455 |  | 0.777 | 0.422 |  | 0.668 | 0.842 |  | >0.999 | 0.997 | 0.997 | 0.997 |
| Neutral valence | | | | | | | | | | | | | | | | | |
| 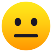 | C1 | 0.057 | 0.057 | 0.974 | 0.726 | 0.853 |  | 0.459 | 0.704 |  | 0.773 | 0.854 |  | 0.988 | 0.988 | 0.988 | 0.988 |
|  | C2 | 0.071 | 0.475 | 0.364 | 0.462 | 0.523 |  | 0.863 | 0.776 |  | 0.933 | 0.229 |  | 0.986 | 0.987 | 0.986 | 0.986 |
|  | C3 | 0.065 | 0.911 | 0.277 | 0.428 | 0.726 |  | 0.433 | 0.786 |  | 0.059 | 0.788 |  | 0.988 | 0.988 | 0.988 | 0.988 |
|  | C4 | 0.144 | **0.017*** | 0.576 | 0.388 | 0.170 |  | 0.057 | 0.959 |  | 0.283 | 0.210 |  | 0.985 | 0.986 | 0.985 | 0.985 |
|  | C5 | 0.109 | **0.042*** | 0.360 | 0.690 | 0.897 |  | 0.665 | 0.580 |  | **0.024*** | 0.281 |  | 0.988 | 0.988 | 0.988 | 0.988 |
|  | C6 | 0.168 | 0.790 | 0.390 | 0.239 | 0.994 |  | 0.812 | 0.991 |  | 0.600 | 0.800 |  | 0.999 | 0.999 | 0.999 | 0.999 |
|  | C7 | 0.067 | 0.576 | 0.256 | 0.251 | 0.240 |  | 0.093 | 0.684 |  | 0.569 | 0.823 |  | 0.988 | 0.988 | 0.988 | 0.989 |
|  | C8 | 0.130 | 0.120 | 0.233 | 0.244 | 0.599 |  | 0.777 | 0.631 |  | **0.033*** | 0.356 |  | 0.986 | 0.985 | 0.985 | 0.985 |
|  | C9 | 0.106 | 0.231 | 0.587 | **0.016*** | 0.051 |  | 0.768 | 0.185 |  | 0.474 | 0.265 |  | 0.984 | 0.985 | 0.985 | 0.985 |
| 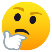 | C3 | 0.056 | 0.692 | 0.836 | 0.081 | 0.836 |  | 0.160 | 0.904 |  | 0.750 | 0.679 |  | 0.992 | 0.992 | 0.992 | 0.992 |
|  | C4 | 0.138 | 0.436 | 0.235 | 0.872 | 0.674 |  | 0.378 | 0.931 |  | **0.002*** | 0.323 |  | 0.992 | 0.992 | 0.993 | 0.992 |
|  | C5 | 0.085 | 0.238 | 0.361 | 0.120 | **0.046*** |  | 0.805 | 0.573 |  | 0.136 | 0.880 |  | 0.993 | 0.993 | 0.993 | 0.993 |
|  | C7 | 0.098 | 0.947 | 0.786 | 0.251 | 0.990 |  | 0.242 | 0.927 |  | 0.758 | 0.452 |  | 0.996 | 0.996 | 0.996 | 0.996 |
|  | C9 | 0.044 | 0.258 | 0.966 | 0.625 | 0.645 |  | 0.735 | 0.648 |  | 0.693 | 0.842 |  | 0.993 | 0.993 | 0.993 | 0.992 |

C1 – crowding, C2 – anterior open bite, C3 – interincisal diastema, C4 – increased overjet, C5 – anterior crossbite, C6 – ideal occlusion, C7 – unilateral posterior crossbite, C8 – anterior open bite plus bilateral posterior crossbite plus crowding, C9 – deep bite.
† Reference category.
* indicates significant association / contribution in the model.
Only *emoji* with a frequency of choice ≥ 10% for a given condition were analyzed.
